# Mobile Subthreshold Exercise Program (MSTEP) for concussion: study protocol for a randomized controlled trial

**DOI:** 10.1186/s13063-022-06239-3

**Published:** 2022-04-26

**Authors:** Sara P. D. Chrisman, Beth J. Bollinger, Jason A. Mendoza, Tonya M. Palermo, Chuan Zhou, M. Alison Brooks, Frederick P. Rivara

**Affiliations:** 1 Center for Child Health, Behavior and Development, Seattle Children’s Research Institute, PO Box 5371, CURE-03, Seattle, WA 98145 USA; 2 Department of Pediatrics, University of Washington, Seattle, USA; 3 Fred Hutchinson Cancer Research Center, Seattle, USA; 4 Department of Anesthesiology and Pain Medicine, University of Washington, Seattle, USA; 5 University of Wisconsin, Madison, USA

**Keywords:** Exercise, Physical activity, Brain concussion, Clinical trial, Child, Randomized controlled trial

## Abstract

**Background:**

Subthreshold exercise, defined as aerobic exercise below the level that causes symptoms, has been utilized as a treatment for youth with persistent postconcussive symptoms (PPCS), but there is currently little evidence to guide use. In addition, prior studies of exercise for PPCS have all required multiple in-person visits. We developed a virtual approach for delivering subthreshold exercise to youth with PPCS called the Mobile Subthreshold Exercise Program (MSTEP), and we have now been funded to conduct a large national randomized controlled trial (RCT) to test its efficacy for reducing concussive symptoms and improving health-related quality of life.

**Methods:**

This investigation is an RCT comparing MSTEP to an active control. We will recruit 200 adolescents 11–18 years old with postconcussive symptoms persisting for at least 1 week but less than 1 year. Youth will be randomized to receive either 6 weeks of subthreshold exercise (MSTEP) or a stretching condition (control). Youth and parents will complete surveys of concussive symptoms at baseline, weekly during the intervention, and at 3 and 6 months. The primary outcomes will be trajectory of concussive symptoms and health-related quality of life over the 6 months of the study. Secondary outcomes will include depression, anxiety, and sleep quality. We will also assess potential mediators of treatment effects including moderate-vigorous physical activity and fear avoidance of concussive symptoms.

**Discussion:**

This multisite RCT of MSTEP will provide vital information regarding the efficacy of a virtually delivered subthreshold exercise program for youth with PPCS, and insight regarding potential mediators of treatment effects, including objectively measured physical activity and fear avoidance of concussive symptoms.

**Trial registration:**

ClinicalTrials.gov NCT04688255. Registered on December 29, 2020.

## Administrative information

Note: the numbers in curly brackets in this protocol refer to SPIRIT checklist item numbers. The order of the items has been modified to group similar items (see http://www.equator-network.org/reporting-guidelines/spirit-2013-statement-defining-standard-protocol-items-for-clinical-trials/).

**Table:** 

Title {1}	Mobile Subthreshold Exercise Program (MSTEP) for Concussion: Study protocol for a randomized controlled trial
Trial registration {2a and 2b}.	ClinicalTrials.gov, NCT04688255. Registered on December 29, 2020
Protocol version {3}	December 9, 2021.
Funding {4}	This work was made possible by a grant from the NICHD, R01HD094722
Author details {5a}	Sara PD Chrisman^1,2^ Beth J Bollinger^1^ Jason A Mendoza^1-3^ Tonya M Palermo^1,4^ Chuan Zhou^1^ M. Alison Brooks^5^ Frederick P Rivara^1,2^ ^1^Seattle Children’s Research Institute, Center for Child Health, Behavior and Development ^2^University of Washington, Department of Pediatrics ^3^Fred Hutchinson Cancer Research Center ^4^University of Washington, Department of Anesthesiology and Pain Medicine ^5^University of Wisconsin, Madison
Name and contact information for the trial sponsor {5b}	Sponsor/ Investigator: SPD Chrisman, MD MPH Center for Child Health, Behavior and Development Seattle Children’s Research Institute, CURE-03, 1920 Terry Avenue, PO Box 5371 Seattle, WA 98145-5005 sara.chrisman@seattlechildrens.org 206-484-2133
Role of sponsor {5c}	The sponsor for this study is the NIH who will have no involvement in the data analysis, interpretation or reporting of results.

## Introduction

### Background and rationale {6a}

Approximately 1.9 million youth sustain a concussion each year [1], and up to 30% experience persistent postconcussive symptoms (PPCS) such as headache, dizziness, and difficulty focusing that continue for weeks or months [2–5]. PPCS results in greater utilization of subspecialty care and can impact immediate and long-term social development, cognitive function, and academic success [6]. Individuals with PPCS often have increased symptoms when engaging in physical activity, and such exertional symptoms can lead to avoidance of physical activity and subsequent disability [6]. Research, including our own work, has examined potential benefit of subthreshold (or rehabilitative) exercise [7–17], with the hypothesis that exercise performed below the threshold that results in symptoms will facilitate recovery. Several randomized controlled trials (RCTs) have been conducted using exercise to treat youth with concussion, showing significant benefit of exercise compared to control [8, 9, 11], but all have required weekly in-person visits, which impedes access for many youth, particularly rural or underserved youth [18–23]. Delivery of physical activity (PA) interventions virtually can help to transcend geographic barriers, thereby improving access [24, 25].

**Fig. 1:**
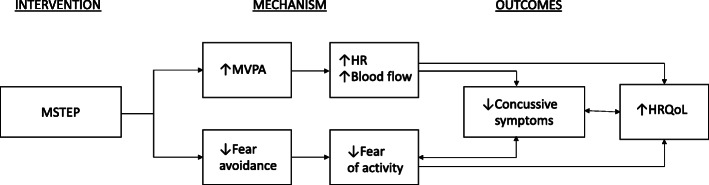
Conceptual model outlining potential mediation of treatment effects in the Mobile Subthreshold Exercise Program (MSTEP) intervention for concussion

Prior human and animal research suggests that postconcussion exertional symptoms may result from dysfunction of cerebral autoregulation, causing transmission of systemic hypertension to the cerebral space [18–20]. Rehabilitative exercise is designed to retrain the body’s physiologic response to exercise, such that an individual is no longer experiencing symptoms with physical activity. In addition, research with rodent models has shown that exercise in the subacute time period following brain injury (> 1 week) increases angiogenesis and concentrations of neurotrophins such as Brain-derived neurotrophic factor (BDNF), which are thought to act as mediators of recovery [26–28]. Parallel research with humans has found increased cerebral blood flow during exercise [29], and associations between aerobic exercise participation and improved cognition in older adults [30, 31]. In our pilot work [16], we found youth with PPCS had a median moderate-vigorous physical activity (MVPA) per day of only 14 min at baseline (measured using accelerometry with hip-mounted ActiGraph GT3X), despite having previously been athletes. This is in contrast to NHANES (National Health and Nutrition Examination Survey) reported average MVPA for US adolescents of 20–45 min per day [32], and guidelines from the US Physical Activity Guidelines Advisory Committee [33] recommending 60 min of MVPA per day for youth. We thus hypothesize that increases in MVPA in part may mediate the improvement in concussive symptoms seen secondary to a rehabilitative exercise approach (Fig. 1).

We also have noted improvement in psychologic factors such as fear avoidance following subthreshold exercise in our prior trials [16, 17], a unique finding given that research on exercise as a treatment for PPCS has primarily focused on physiologic change rather than psychological factors [18]. Fear avoidance is a concept that has been used to understand the development and perpetuation of chronic pain in adults and youth [34–36]. According to fear avoidance models, individuals experiencing pain can develop high levels pain-catastrophizing, or a tendency to magnify and ruminate on the potential threat associated with pain and discomfort, leading to feelings of helplessness [37]. Pain-catastrophizing provokes a fear of anticipated pain, avoidance of activities that might cause pain, and ultimately inactivity and dysfunction. We have applied this model to concussion [16, 17], theorizing that individuals with concussion may become fearful that any discomfort during PA is indicative of worsening injury, and begin to avoid all PA. Avoiding all PA then creates functional disability, as youth develop neurophysiologic changes due to lack of movement [38], and secondary mood symptoms due to the disconnection from their normal activities (e.g., school and sports) and peers [6]. Our pilot data indicated declines in fear avoidance that parallel declines in concussive symptoms [16].

We also frame our understanding of the pain experience using the biopsychosocial model, which posits that an individual’s experience of symptoms such as pain is influenced by a complex array of psychosocial factors, particularly individual characteristics (such as gender) and parent and family behaviors [39, 40]. It is thus essential that we explore potential differential response to a rehabilitative exercise intervention in view of these factors. We have made efforts to include sex in our analytic models, as females are diagnosed with concussions more frequently than males playing the same sport [41, 42] and are more likely than males to experience PPCS [43–46], but males have greater responses (i.e., increases in MVPA) to exercise interventions when utilized in contexts such as obesity [47, 48]. No prior studies of rehabilitative exercise interventions for concussion have examined differential effects by sex [18, 19, 21, 23, 49]. Parental behavior should also be considered, as parents play an important role in the social context of pain and discomfort, and parental concern about the potential danger associated with pain can increase the likelihood of youth concern [50]. Research suggests that parental protective or solicitous behavior (such as paying greater attention to a child due to pain) is associated with impairments in function [51–53]. It is critical to account for biopsychosocial factors in our analysis and specifically explore whether sex or parental protectiveness result in differential responses to rehabilitative exercise for PPCS.

In sum, we are undertaking a large multisite RCT of a virtually delivered subthreshold exercise program (MSTEP) to treat youth with PPCS. We will also test an innovative conceptual model to explain the MSTEP treatment effect, including both physiologic and psychological mediators (Fig. 1). Sustained increases in heart rate due to MVPA produce increased cerebral perfusion [54], which might underlie decreased symptoms and improved function. Encouraging youth to exercise despite fears of exacerbating symptoms could also result in lessened activity avoidance, thus improving function [55]. Given the influence of parent and family factors, we will also assess the role of parental behavior in shaping youth response to the intervention.

### Objectives {7}

The goal of this study is to assess the efficacy of a virtually delivered subthreshold exercise program (MSTEP) for treating youth with PPCS. We will test the effect of 6 weeks of MSTEP on trajectory of concussive symptoms and health-related quality of life (HRQoL) 6 months after study entry compared to youth in an active control group (stretching). We will also examine mediation of treatment effects by changes in physiologic mediators (MVPA) and psychological mediators (fear avoidance of concussive symptoms).

The primary hypothesis of this RCT is that MSTEP will improve outcomes (concussive symptoms and HRQoL) in youth with concussion compared to a control intervention. We further hypothesize that improvements in outcomes due to MSTEP will be mediated by increases in aerobic activity (i.e., MVPA) and decreases in fear avoidance of concussive symptoms.

### Trial design {8}

Youth with PPCS will be randomized to 6 weeks of either MSTEP (a virtually delivered subthreshold exercise program) or an active control (stretching). This is a superiority trial, testing whether MSTEP is superior to the active control. Randomization will be allocated 1:1 to MSTEP and control, stratified by severity of concussive symptoms and sex at birth. Data will be collected at baseline, weekly during the intervention and at 6 weeks, 3 months, and 6 months.

## Methods: participants, interventions, and outcomes

### Study setting {9}

Subjects will be recruited from pediatric subspecialty clinics in Sports Medicine and Rehabilitation Medicine in Seattle, WA, and Madison, WI, in the USA. In addition to those two locations, multiple avenues will be utilized for broader national recruitment including clinician referral and online advertisement.

### Eligibility criteria {10}

#### Inclusion criteria

1. 11–18 years old
2. Concussion occurring 1 week to 12 months prior to the start of the study and diagnosed by a clinician trained in concussion management consistent with the 2017 Berlin consensus definition of concussion (“A traumatic brain injury, induced by biomechanical forces”)
3. Continuing to exhibit at least 3 symptoms at baseline with a total score on the Health and Behavior Inventory (HBI) of at least 10

#### Exclusion criteria

1. Youth not fluent in English or at least one parent not fluent in English or Spanish
2. Other injuries or medical conditions in addition to concussion that prompted a clinician to recommend against MVPA
3. Youth who indicate they are completing an average of 30 min per day or greater of physical activity that increases their heart rate (indicative of a minimal need for a physical activity intervention)
4. Youth who are engaging with a physical therapist to increase aerobic activity
5. Youth who have been fully cleared for sport

### Who will take informed consent? {26a}

Research coordinators will obtain verbal consent from parents and youth via phone or video conferencing using a standardized interview documenting risks and benefits of the study. Parents and youth will document consent by electronically signing parent permission and assent or consent forms. All procedures have been approved by the Seattle Children’s Institutional Review Board.

### Additional consent provisions for collection and use of participant data and biological specimens {26b}

Not applicable as biologic specimens are not collected for this study.

## Interventions

### Explanation for the choice of comparators {6b}

We have developed a novel approach to deliver a subthreshold exercise intervention to youth with concussion (Mobile Subthreshold Exercise Program, MSTEP) that utilizes video conferencing software and personal fitness devices such that the intervention can be delivered completely remotely. We will compare this intervention to an active control (stretching) to ensure engagement with the study by control participants.

### Intervention description {11a}

#### Mobile-delivered subthreshold exercise (MSTEP)

The MSTEP intervention consists of aerobic exercise delivered at a subsymptom level (i.e., an intensity that does not provoke symptoms) and then is slowly advanced over a period of 6 weeks. Exercise engagement is supported by (1) video conferencing weekly with health coaches to set goals and (2) physical activity trackers to measure activity level and heart rate (Fitbit). Youth will be instructed to exercise at home daily with an initial goal of achieving light physical activity (LPA) for 10 min per day with a heart rate (HR) of 120 beats per minute (bpm). They will be asked to guide the intensity of their exercise using symptoms, decreasing the intensity of exercise sessions if symptoms worsen. Intensity will be advanced as tolerated until they are achieving MVPA, at HRs of 140–150 bpm. Youth who are not experiencing symptoms will be advanced in their HR goals by 10 bpm every few days, until they reach a HR goal of 140–150 bpm. Once they are able to tolerate PA at 140–150 bpm for 10 min, they will be advanced in duration until meeting minimum US recommendations for daily MVPA, 60 min/day [33], after which they will be encouraged to exercise ad libitum above 60 min/day of MVPA. Participants will be allowed to choose the type of exercise, and examples will be provided as needed. They will also continue to receive usual care from their concussion providers. We have piloted these methods previously, finding excellent feasibility and acceptability [17].

#### Active control (stretching)

We chose to utilize an active control to (1) provide a measure of blinding (participants will be told they will receive one of two exercise interventions) and (2) improve retention in the control group. Control subjects will be provided a series of stretching exercises, which will also advance throughout the 6 weeks. Subjects will initially be given two stretches, primarily focused on the neck and upper back (e.g., cervical retraction, scapular retraction). Additional stretches will be added through weekly discussions with the health coach if subjects are successfully tolerating and completing initial stretches, adding approximately one new stretching exercise per week. Control subjects will also continue to receive usual care from their concussion providers. We have also successfully piloted this approach previously [16].

### Criteria for discontinuing or modifying allocated interventions {11b}

Youth will be free to discontinue participation at any time. We do not have criteria for investigator initiated discontinuation of treatment.

### Strategies to improve adherence to interventions {11c}

#### Frequent contact with study staff via video conferencing

Youth in both groups will be contacted by the research staff weekly via HIPAA-compliant video conferencing to discuss barriers and facilitators to completing exercises, and assess frequency and severity of symptoms. The use of video conferencing was successful as a means of enhancing participant engagement in our prior studies in which we retained > 90% of intervention and control participants to 12-month follow-up [56].

#### Use of physical activity trackers

Utilizing the Fitbit provides participants with a visible cue to gauge their progress towards their activity goals (heart rate and duration). Prior studies have suggested that the use of PA trackers results in increased physical activity, particularly when paired with study staff contact [57, 58].

#### Self-report of exercise frequency to increase monitoring

Participants in both arms will complete daily surveys regarding days of exercise completed per week to both provide a prompt to complete exercises and measure fidelity to intervention arm.

#### Visual cues

Youth in both arms will be provided a water bottle with the study logo and a calendar with their daily schedule as reminders to complete their exercise program.

### Relevant concomitant care permitted or prohibited during the trial {11d}

Participants in both arms will continue to receive usual care from their concussion provider.

### Provisions for post-trial care {30}

No post-trial care is provided.

### Outcomes {12}

#### Primary outcome measures

##### Concussive symptoms

Concussive symptoms will be measured using the Health Behavior Inventory (HBI). The HBI is a component of the NIH common data elements (CDEs) for research on concussion [59, 60], and is also contained in the Sport Concussion Assessment Tool-5 Child Version (Child-SCAT5) [61]. It is a 20-item instrument that measures postconcussive symptoms on a 4-point Likert scale, yielding scores in somatic and cognitive domains demonstrated by factor analysis to be robust across raters and time (Cronbach’s alpha = 0.85–0.94) [62]. The HBI has demonstrated validity and reliability among adolescents and individuals with mild TBI [62–66]. Higher scores indicate greater severity. HBI will be assessed at baseline, weekly during the intervention period (6 weeks) and at 3 months and 6 months (9 time points), see SPIRIT figure for details.

##### Health-related quality of life (HRQoL)

HRQoL will be measured using Pediatric Quality of Life Inventory (PedsQL). PedsQL is a 23-item scale that measures health-related quality of life. It covers four domains of physical, emotional, social, and school function on a 5-point Likert scale with higher scores indicating greater quality of life. PedsQL asks respondents how much of a problem each item has been over the past month on a scale ranging from “never” to “almost always.” We will use youth- and parent-proxy versions, both of which have established validity and reliability (Cronbach’s alpha = 0.88 for child and 0.90 for parent report) [67–70]. PedsQL has been successfully used in youth TBI research previously by our team of investigators and others [71–75]. Higher scores indicate better functioning. PedsQL will be assessed at baseline, 6 weeks, 3 months, and 6 months (4 time points).

#### Secondary outcome measures:

##### Depression

Depressive symptoms will be measured using the Patient Health Questionnaire-9 (PHQ-9), a component of the NIH CDEs for research on concussion. It is a 9-item instrument that measures depressive symptoms on a 4-point Likert scale with higher scores indicating greater severity. This scale has demonstrated validity and reliability among adolescents and individuals with mild TBI [76–80], with internal consistency reported as 0.86 in adolescents with TBI [72]*.* PHQ-9 will be assessed at baseline, 6 weeks, 3 months, and 6 months (4 time points).

##### Anxiety

Anxiety symptoms will be measured with the Generalized Anxiety Disorder-7 item scale (GAD-7), a 7-item standardized anxiety measure that asks youth to rate how often they have been bothered by anxiety symptoms using a 4-point Likert scale. It has been shown to have good reliability, as well as criterion, construct, factorial, and procedural validity for assessing anxiety [81, 82]. GAD-7 will be assessed at baseline, 6 weeks, 3 months, and 6 months (4 time points).

##### Sleep quality

Sleep quality will be measured with the short form version of the Adolescent Sleep Wake Scale (ASWS-10), a 10-item scale designed to capture sleep quality including difficulties with sleep onset and maintenance in youth (Cronbach’s alphas 0.80–0.86) [83]. ASWS-10 will be assessed at baseline, 6 weeks, 3 months, and 6 months (4 time points).

#### Potential mediators:

##### Fear of concussive symptoms

We will measure youth emotional reactions to concussive symptoms by adapting the Fear of Pain Questionnaire (FOPQ), parent-proxy and child report, to pertain to concussive symptoms. The FOPQ has good reliability and validity for measuring pain-related fear in youth (Cronbach’s alpha 0.92) [84], and we adapted this scale to concussion by replacing “pain” with “concussive symptoms.” Example items include the following: “When I feel concussive symptoms, I am afraid that something terrible will happen” and “I can’t do all the things normal people do because it’s so easy to hurt my body.” FOPQ will be assessed at baseline, 6 weeks, 3 months, and 6 months (4 time points).

##### Moderate-to-vigorous physical activity (MVPA)

We will measure MVPA using the ActiGraph GT3X Link (ActiGraph LLC, Pensacola, FL) worn at the hip with a belt or clip with methodology refined in our previous studies [16, 17, 85–88]. In line with current standards, we will program the GT3X accelerometers to collect raw data at a sample frequency of 30 Hz, processed into 1-s epochs [89–91]. We will use the following accelerometer data quality standards including criteria for: (1) nonwear and wear time as validated by Choi and colleagues [92] and (2) valid days (10 or more hours of accelerometer wear/day) as established by Trost and colleagues [32, 93–95]. Youth will be asked to wear the accelerometers for 7 days, and at least 4-valid days of accelerometer data will be used in analyses to estimate habitual physical activity [91]. Youth will re-wear accelerometers if they have less than 4-valid days of data. We will use the accelerometer cutpoints for LPA and MVPA developed by Evenson and colleagues [96], which have the highest classification accuracy [97]. Total minutes above the respective thresholds will be divided by the number of valid days to obtain minutes of LPA and MVPA per day, the latter of which will serve as the main physical activity variable. Similar methods will be used to characterize daily sedentary behavior (SED) [32], i.e., minutes below the cutpoint for LPA, as a potential exploratory variable. Accelerometry will be completed at 3 time points, baseline, 6 weeks, and 3 months (each for 1 week’s time).

#### Covariates

##### Demographics and medical history

Youth and parents will complete surveys regarding demographics (NIH common data elements (CDEs) of gender, race/ ethnicity, socioeconomic status, age, height and weight) and concussion history. Demographics will be collected at baseline only.

##### Parental protectiveness

We will measure parental protectiveness using the Adult Responses to Children’s Symptoms (ARCS), adapted for concussive symptoms: The ARCS is a 29-item 0–4 Likert questionnaire that assess parental responses to youth symptoms and includes 4 sub-scales: protective, minimizing, distracting, and monitoring. We adapted this to concussion by replacing “pain” with “concussive symptoms” [52, 53]. ARCS will be collected at baseline only.

Participant timeline {13}

Participant timeline is shown in Table 1.

**Table 1:** Schedule of enrolment, allocation and study assessments for the Mobile Subthreshold Exercise Program (MSTEP) study for concussion

TIMEPOINT	Study period									
	Enrolment	Allocation	Intervention period (weeks)						Follow-up (months)	
	***− t***_***1***_	0	1	2	3	4	5	6	***3***	***6***
**ENROLMENT:**										
Eligibility screen	X									
Informed consent	X									
**Allocation**		X								
**INTERVENTIONS:**										
***MSTEP***										
***CONTROL***										
**ASSESSMENTS:**										
**Covariates**										
Parental protectiveness (ARCS)	X									
Demographics and medical history	X									
**Primary outcomes**										
*Concussive symptoms, (HBI)*	X		X	X	X	X	X	X	X	X
*Health-related quality of life (PedsQL)*	X		X	X	X	X	X	X	X	X
**Secondary outcomes**										
*Depression* *(PHQ9)*	X							X	X	X
*Anxiety* *(GAD7)*	X							X	X	X
*Sleep quality* *(ASWS-10)*	X							X	X	X
**Mediators**										
*MVPA*^*g*^ *(accelerometry)*	X							X	X	
*Fear avoidance (FOPQ)*	X							X	X	X

*ARCS* adult responses to children’s symptoms, *HBI* Health and Behavior Inventory, *PedsQL* Pediatric Quality of Life, *PHQ-9* Patient Health Questionnaire-9, *GAD7* Generalized Anxiety Disorder-7, *ASWS-10* Adolescent Sleep Wake Scale-10, *MVPA* moderate-vigorous physical activity, *FOPQ* Fear of Pain questionnaire

### Sample size {14}

We plan to recruit 200 subjects. Given the RCT design and repeated assessments, we calculate power for our primary outcomes (HBI) and (PedsQL) using two-way repeated analysis of variance (ANOVA) with two factors: study group is the between-subject factor, and time point (baseline, 6 weeks, 3 months, and 6 months) is the within-subject factor. To address our main hypothesis of differences in trajectories, we will test the between-within interaction effect, i.e., the interaction between study group and time. If we were to see similar differences in means between the two study groups on HBI and PedsQL as we did in our pilot STEP RCT [16], our planned sample size of 200 would provide > 99% power to detect a significant group-by-time interaction (i.e., differences in trends over time between the groups) in both HBI and PedsQL. This estimate is based on 4 time points, similar but slightly larger variances as we observed in pilot data, and an ICC = 0.5 for moderate within-subject correlations. We also wanted to ensure we would have sufficient power to assess mediation. For this power calculation, we used the regression model approach for single mediator. Vittinghoff [98] showed that for the linear regression, ${y}_i={b}_0+{b}_1{x}_i+{b}_2{m}_i+{\epsilon}_i,{\epsilon}_i\sim N\left(0,{\sigma}_e^2\right),$ testing the mediation effect is equivalent to testing the null hypothesis *b*_2_ = 0 versus the alternative hypothesis *b*_2_ ≠ 0. The sample size depends on power, type I error rate, regression coefficient *b*_2_, standard deviation of the mediator *σ*_*m*_, standard deviation of the error term in the above regression *σ*_*e*_, and the correlation between the predictor *x* and the mediator *m, ρ*_*xm*_. Using residualized change scores from our pilot data for the outcome (HBI) and mediators (MVPA and FOPQ) [16], we found that if we were to observe similar mediation effects from MVPA levels and FOPQ as in our pilot data, our proposed total sample size of 200 would provide 78% power to assess mediation by MVPA and 95% power to assess mediation by FOPQ.

### Recruitment {15}

Patients will be recruited from both local clinic populations and nationally via contact with colleagues who care for youth with concussion and online advertisements.

## Assignment of interventions: allocation

### Sequence generation {16a}

We will utilize block randomization, stratified by severity of concussive symptoms (HBI ≥ 20) and sex at birth, with computer-generated random number sequences.

### Concealment mechanism {16b}

The randomization table will not be visible to the research staff.

### Implementation {16c}

Allocation will occur via REDCap.

## Assignment of interventions: Blinding

### Who will be blinded {17a}

We will describe the study as a comparison between exercise programs, in order to blind participants as to whether they are in the intervention or control arm. The statistician will be blinded to intervention status by labeling the intervention variable non-specifically in the dataset (i.e., 1 and 2). The PI and research coordinators will not be blinded.

Procedure for unblinding if needed {17b}

Not applicable as this is not a pharmaceutical trial.

## Data collection and management

### Plans for assessment and collection of outcomes {18a}

We will collect two types of data: [1] survey data and [2] physical activity data via accelerometry. Please see Table 1 for time points of data collection.

Survey data will be collected online using REDCap and self-report.

Accelerometry data will be collected using the ActiGraph GT3X Link (see section “Outcomes {12}”).

### Plans to promote participant retention and complete follow-up {18b}

Please see section “Strategies to improve adherence to interventions {11c}” as this section was written to pertain both to adherence and retention.

### Data management {19}

Survey data will be stored online in REDCap. When possible, data fields will be limited to ensure data quality (e.g., utilizing ranges for dates). Accelerometry data will be downloaded from actigraphs and merged with other data using unique identifiers.

### Confidentiality {27}

Survey data will be entered and stored in a HIPAA-compliant online database (REDCap) accessed only by study staff. Data from accelerometry will be stored on a server at the Seattle Children’s Research Institute with strictly controlled physical access both at the perimeter and at building ingress points by professional security staff utilizing video surveillance, state of the art intrusion detection systems, and other electronic means for ensuring confidentiality. After data is collected, information which would identify the subjects will be removed and code numbers used instead with the translation between the code and PHI stored in a separate file. All analyses will be conducted with deidentified data.

### Plans for collection, laboratory evaluation, and storage of biological specimens for genetic or molecular analysis in this trial/future use {33}

These are not applicable as these types of data are not being collected.

## Statistical methods

### Statistical methods for primary and secondary outcomes {20a}

The primary outcome analyses will be assessed using intention-to-treat with the predictor of interest being experimental group regardless of adherence with assigned treatment. We will examine the impact of adherence on treatment effects in secondary analyses. Pre-identified potential confounders such as demographics (age, sex, and body mass index [BMI]), baseline biopsychosocial factors (standardized measures of depression and anxiety [PHQ-9 and GAD-7]), baseline clinical factors (history of prior concussion, severity of symptoms, duration of symptoms at entry into the study, outcome measures at baseline), and parental protective behavior (Adult Responses to Children’s Symptoms, ARCS) will be summarized using descriptive statistics. In addition to stratification factors (age, sex, and severity of symptoms), we make the a priori decision to control for biopsychosocial factors, particularly parental protective behavior, as well as any other unbalanced confounders from the list above as independent predictors in all subsequent regression analyses. We will model and compare trajectories of outcome measures using mixed effects models. We choose mixed effects modeling for its ability to account for variation at different levels, flexibility regarding time trend and unequally spaced time points, and ease with management of missing data.

The first part of the primary analysis will examine differences in HBI and PedsQL (primary outcomes) between study arms post-intervention (6 weeks) and follow-up (6 months) respectively. The main analytic tool will be based on covariate-adjusted regression models. The two primary outcomes can be considered as continuous outcomes thus linear regression models will be applied, in which group assignment will be the predictor of interest and other pre-identified confounders will be adjusted as covariates.

The second part of the primary analysis will examine trajectories of concussion symptoms. Given that concussive symptoms (HBI) will be assessed 9 times, we believe the data will provide sufficient resolution to model time as a continuous variable when examining change in HBI over time. Let *Y*_*ij*_ denote the HBI measured for *i*th subject at time *t*_*j*_, *x*_*i*_ denote the group assignment for *i*th subject, and ***Z***_*i*_ denote the vector of covariates; we will consider the following general model specification: $(1)\ {Y}_{ij}=f\left({\boldsymbol{\beta}}_i^{\left({x}_i\right)},{t}_j\right)+\boldsymbol{\gamma} {\boldsymbol{Z}}_i+{\epsilon}_{ij};{\boldsymbol{\beta}}_i^{\left({x}_i\right)}\sim N\left({\boldsymbol{b}}^{\left({x}_i\right)},\boldsymbol{\Sigma} \right);{\epsilon}_{ij}\sim N\left(0,{\sigma}^2\right)$. Function *f*(***β***, *t*) can take flexible functional forms to capture different shapes of trajectories. For example, *f*(***β***, *t*) = *β*_0_ + *β*_1_*t* represents a linear trend over time; *f*(***β***, *t*) = *β*_0_ + *β*_1_*t* + *β*_2_*t*^2^ represents a quadratic trend over time; or more generally, we can assume a restricted cubic spline for *f*(***β***, *t*) to represent more flexible trajectory shapes. Based on our pilot study and prior studies in the literature, we have observed that concussive symptoms and other health outcomes exhibit faster rates of improvement in the acute phase post-injury (< 3 months), with rates then decreasing and leveling off. For these reasons, a more parsimonious and accurate function for time trend could be exponential decay function in the form *f*(***β***, *t*) = *β*_2_ + (*β*_0_ − *β*_2_) × exp(−*β*_1_*t*),where *t* is time, *β*_0_ is the initial value at *t = 0*, *β*_2_ is the asymptote as *t* goes to infinity, *β*_1_, *constrained to be positive*, is the rate of change or how quickly the process decays from the initial value to the asymptote [99]. Parameters ***b***^(1)^ and ***b***^(2)^ are the group specific shape parameters, and equality of these parameters will be tested using multivariate Wald test to assess the hypothesis that concussive symptoms (HBI) decline more quickly in the MSTEP group compared to control. Note the rate of change *β*_1_ could also be influenced by other biopsychosocial variables. We can address this by considering a more advanced hierarchical model in which: [2] log(*β*_1_(*x*_*i*_, ***Z***_*i*_)) = *α*_0_ + *α*_1_*x*_1_ + ***α***_2_***Z***_*i*_, the logarithmic transformation ensures positivity of the change rate and parameter *α*_1_captures the difference in rate of change between study arms.

Health-related quality of life as measured by PedsQL will be assessed at 4 time points. The smaller number of time points limits options for trajectory shapes; thus constraining us to examine changes over time between groups by modeling time as a discrete predictor: (3) *Y*_*ij*_ = *β*_0*i*_ + *b*_1_*x*_*i*_ + *b*_2*j*_*t*_*j*_ + *b*_3*j*_*x*_*i*_ × *t*_*j*_ + *ϵ*_*ij*_; *β*_0*i*_~*N*(*b*_0_, *τ*^2^); *ϵ*_*ij*_~*N*(0, *σ*^2^).

With this specification, the random intercept *β*_0*i*_ captures within-subject correlation, and coefficients for the group-by-time interactions *b*_3*j*_ estimate the differences in changes over time between groups. Individual coefficients can be tested using Wald test, and overall group-by-time interaction can be tested using likelihood ratio test.

### Interim analyses {21b}

This is not applicable as this is not a pharmaceutical trial.

### Methods for additional analyses (e.g., subgroup analyses) {20b}

We will examine potential mediation of the intervention effect by [1] MVPA and [2] fear avoidance of concussive symptoms We plan to conduct two sets of mediation analyses. The first set of mediation analyses will focus on the changes between baseline and 3 months. The main motivation for this choice is that this analysis amounts to a conventional single mediator model which is easy to implement and has a simple interpretation, directly addressing questions such as, “How much of the effect of MSTEP on improved concussive symptoms is mediated through increased MVPA or decreased fear avoidance?” We will use residualized change scores instead of the difference scores in part because they adjust for baseline differences and avoid some of the problems with the reliability of difference scores. The residualized change score is the difference between the observed score at 3 months and predicted score at 3 months, where baseline score is used to predict 3-month score. With residualized change scores calculated for mediators (MVPA and FOPQ) and outcomes (HBI, PedsQL), we can proceed to the standard single mediator model as depicted in the following figure. The mediation model can be represented using the three ordinary-least-squares (OLS) regression equation: *Y*_*i*_ = *i*_1_ + *cX*_*i*_ + *ϵ*_1*i*_; *M*_*i*_ = *i*_2_ + *aX*_*i*_ + *ϵ*_2*i*_; *Y*_*i*_ = *i*_3_ + *c*^′^*X*_*i*_ + *bM*_*i*_ + *ϵ*_3*i*_, where *c* is the total effect of *X* on *Y*, *c’* is the direct effect of *X* on *Y* controlling for *M*, *a* is the effect of *X* on *M*, *b* is the effect of *M* on *Y* controlling for *X*, and *ab* is the mediated effect. Significance of mediated (indirect) effect can be tested via Sobel test or bootstrap methods [100].

The second set of mediation analyses will be longitudinal mediation using a latent growth curve modeling (LGCM) framework, allowing us to utilize the repeated assessments and therefore provide more insight into the mediation process, while allowing more accurate functional forms. Step 1: The growth trajectories of mediator and outcome are modeled separately to determine the trajectory shapes and estimate the overall changes in the mediator and the outcome. In the following, we assume exponential decay trajectories for both mediator process and outcome process, although different forms can be assumed. The measurement models can be specified as: ***Mediator Process :*** *M*_*ij*_ = *β*_2*i*_ + (*β*_0*i*_ − *β*_2*i*_) exp(−*β*_1*i*_*t*_*j*_) + *e*_*ij*_

$$ \boldsymbol{Outcome}\ \boldsymbol{process}:{Y}_{ij}={\gamma}_{2i}+\left({\gamma}_{0i}-{\gamma}_{2i}\right)\exp \left(-{\gamma}_{1i}{t}_j\right)+{e}_{ij} $$

Step 2: Mediational process is then modeled by combining the two growth curve models and relating the growth factors (random effects *β*’s and *γ*’s) and the independent variable *X* (group assignment) in the structural model expressed as follows:

$$ {\beta}_{0i}={b}_0+{a}_0{X}_i+{\xi}_{0i} $$

$$ {\beta}_{1i}={b}_1+{\eta}_2{\gamma}_{0i}+{a}_1{X}_i+{\xi}_{1i} $$

$$ {\beta}_{2i}={b}_2+{\eta}_3{\gamma}_{0i}+{a}_2{X}_i+{\xi}_{2i} $$

$$ {\gamma}_{0i}={g}_0+{c}_0{X}_i+{\epsilon}_{0i} $$

$$ {\gamma}_{1i}={g}_1+{\eta}_4{\beta}_{0i}+{\eta}_5{\beta}_{1i}+{\eta}_6{\beta}_{2i}+{c}_1{X}_i+{\epsilon}_{1i} $$

$$ {\gamma}_{2i}={g}_2+{\eta}_7{\beta}_{0i}+{\eta}_8{\beta}_{1i}+{\eta}_9{\beta}_{2i}+{c}_2{X}_i+{\epsilon}_{2i} $$

These equations posit that [1] the initial state of outcome process (*γ*_0_) affects rate of change (*β*_1_) and asymptote (*β*_2_) of the mediator process [2]; initial state, rate of change, and asymptote of moderator process affect both rate of change and asymptote of the outcome process. The single mediator model and longitudinal mediation model using LGCM will be implemented estimated using M-Plus and R statistical software.

### Methods in analysis to handle protocol non-adherence and any statistical methods to handle missing data {20c}

Multiple imputation (MI) will be used to adjust model estimates that include incomplete data. Specifically, we will generate multiple imputed datasets with missing values filled in using chained equations (MICE). Baseline characteristics will be imputed using predictive mean matching and missing outcomes will be imputed using joint linear mixed models. Regression models will be fitted to each imputed dataset and the results will be pooled to produce the final estimates for inference.

### Plans to give access to the full protocol, participant-level data, and statistical code {31c}

We will upload the final protocol to clinicaltrials.gov once all data has been collected. Deidentified data and statistical code will be made available to qualified investigators upon request.

## Oversight and monitoring

### Composition of the coordinating center and trial steering committee {5d}

The PI (SC) will oversee the research team and ensure all aspects of the protocol are followed. The PI will also run monthly meetings with all co-investigators (FP, TP, CZ, JM, MAB) to provide additional oversight regarding any issues that arise. A Data Safety Monitoring Board (DSMB) has also been developed consisting of a pediatrician specialized in Adolescent Medicine, a rehabilitation medicine scientist with expertise in concussion clinical care and research, and a senior research scientist with expertise in behavior change research regarding physical activity.

### Composition of the data monitoring committee, its role and reporting structure {21a}

The PI (SC) will oversee data monitoring to assess issues with missingness and ensure quality. All analysis will be completed by the study statistician (CZ). A DSMB has also been developed as described above.

### Adverse event reporting and harms {22}

Adverse events will be reported as per IRB and NIH guidelines. We will also utilize a data safety monitoring board (DSMB) who will address any safety or ethical concerns. Prior to the start of protocol use, the DSMB reviewed the study procedures and plans for safety monitoring. Over the course of the trial, the board will review the data collection procedures at multiple time points (approximately twice per year, depending on recruitment and issues that arise) and will monitor the occurrence of any adverse events. All potentially adverse events will be reported to the DSMB within 48 h of occurrence. These adverse events include deaths, suicide attempts, severe side effects from physical activity, study dropout, hospitalizations, and/or clinical deterioration defined as the development of new suicidal or homicidal behaviors.

Each year, the DSMB will receive a report that summarizes all serious and unexpected adverse events and the DSMB will produce a report that reviews and summarizes [1] the committee’s opinion as to the safety, confidentiality, and privacy of the study and if study investigators are adequately assuring safety, confidentiality, and privacy [2]; a progress summary towards recruitment and follow-up goals [3]; any concerns relating to serious or unexpected adverse events. This yearly summary will be forwarded to the principal investigators, who will submit it to the Seattle Children’s Research Institute IRB.

### Frequency and plans for auditing trial conduct {23}

The PI will lead study meetings each week with the research staff and will monitor for any concerns. The DSMB will meet approximately biannually, or as needs dictate.

### Plans for communicating important protocol amendments to relevant parties (e.g., trial participants, ethical committees) {25}

Changes in protocol will be communicated to the public via clinicaltrials.gov. If participants are actively engaged in the study, they will be notified of any changes that would affect their participation.

### Dissemination plans {31a}

Results from the proposed project will be disseminated widely through traditional pathways, first through conference presentations targeting academicians, primary care providers, and concussion specialists, and then via peer-reviewed publications in academic journals. Dissemination to community stakeholders will occur through local and national presentations. We will also work with Seattle Children’s Research Institute media channels to promote publications and presentations that arise from this work.

## Discussion

The MSTEP study is the largest study to date to evaluate the impact of subthreshold exercise on youth with concussion, and the only one to deliver an exercise intervention completely virtually. This study builds on several years of work [15–17], including exploration into the feasibility and acceptability of this approach, and pilot data regarding potential benefit for youth with concussion. Our team began by studying subthreshold exercise as it is delivered clinically at our institution [15], then worked to translate this intervention into a research space [16], and finally adapted our methodology such that the intervention could be delivered completely virtually [17]. We are now testing this approach in a randomized controlled trial with a large sample size, and will be able to examine not only intervention effects, but variation between subjects and across demographic factors.

The MSTEP study is also unique in that it has been powered to examine potential mechanisms underlying treatment effects, particularly whether increases in MVPA are responsible for benefit [9] or whether other processes may matter more, such as decreases in fear of concussive symptoms [16]. Exploring mediators of treatment effects is key to optimizing these approaches in the future, as this information can guide our decisions regarding how to amplify treatments in future work.

Prior studies of subthreshold exercise interventions required intensive in-person visits weekly over several months [8, 9, 11]. Removing the need for in-person visits and converting the intervention to a virtual space improves access to youth who are distant from a medical center and is thus essential for ensuring equitable access to treatment. Developing a virtual intervention also ensures that exercise interventions can be available to treat youth with concussion during times when in-person visits present greater risk, such as during a pandemic. We are excited to test this approach with a broader sample of youth to determine whether treatment effects remain robust as we scale up these methods.

## Conclusions

Subthreshold exercise interventions have been shown to be effective for youth with concussion [9, 10], but the approaches utilized require extensive in-person visits which impedes access for many youth, particularly those distant from a major medical center or those concerned about undertaking an in-person visit due to risk of infection. We are testing the efficacy of a virtually delivered subthreshold exercise program for youth with concussion (MSTEP), using HIPAA-compliant video conferencing and physical activity tracking. Findings from the MSTEP study will be of great interest to clinicians caring for youth with concussion and may lead to changes in guidelines of care.

Trial status

Date recruitment began: 3/22/21

Estimated date of recruitment end: 4/15/25

Protocol version 8

## Abbreviations

MSTEP: Mobile Subthreshold Exercise Program
PPCS: Persistent postconcussive symptoms
RCTs: Randomized controlled trials
BDNF: Brain-derived neurotrophic factor
PA: Physical activity
MVPA: Moderate-vigorous physical activity
REDCap: Research Electronic Data Capture
HIPAA: Health Insurance Portability and Accountability Act
NHANES: National Health and Nutrition Examination Survey
HRQoL: Health-related quality of life
HR: Heart rate
BPM: Beats per minute
HBI: Health and Behavior Inventory
LPA: Light physical activity
CDEs: Common Data Elements
SCAT5: Sport Concussion Assessment Tool, version 5
PedsQL: Pediatric Quality of Life Inventory
GAD7: Generalized Anxiety Disorder-7 item
PHQ9: Patient Health Questionnaire-9 item
ARCS: Adult Responses to Children’s Symptoms
ASWS10: Adolescent Sleep Wake Scale-10 item
FOPQ: Fear of Pain Questionnaire
ANOVA: Analysis of variance
SED: Sedentary behavior
BMI: Body mass index
LGCM: Latent growth curve modeling
MI: Multiple imputation
DSMB: Data Safety Monitoring Board
